# H-NS is a repressor of major virulence gene loci in *Vibrio parahaemolyticus*

**DOI:** 10.3389/fmicb.2014.00675

**Published:** 2014-12-12

**Authors:** Fengjun Sun, Yiquan Zhang, Yefeng Qiu, Huiying Yang, Wenhui Yang, Zhe Yin, Jie Wang, Ruifu Yang, Peiyuan Xia, Dongsheng Zhou

**Affiliations:** ^1^Department of Pharmacy, Southwest Hospital, Third Military Medical UniversityChongqing, China; ^2^State Key Laboratory of Pathogen and Biosecurity, Department of Biosafety, Beijing Institute of Microbiology and EpidemiologyBeijing, China; ^3^School of Medicine, Jiangsu UniversityZhenjiang, China; ^4^Laboratory Animal Center, Academy of Military Medical SciencesBeijing, China

**Keywords:** *Vibrio parahaemolyticus*, H-NS, T3SS1, Vp-PAI, T6SS2

## Abstract

*Vibrio parahaemolyticus*, a leading cause of seafood-associated diarrhea and gastroenteritis, harbors three major virulence gene loci T3SS1, Vp-PAI (T3SS1+*tdh2*) and T6SS2. As showing in this study, the nucleoid-associated DNA-binding regulator H-NS binds to multiple promoter-proximal regions in each of the above three loci to repress their transcription, and moreover H-NS inhibits the cytotoxicitiy, enterotoxicity, hemolytic activity, and mouse lethality of *V. parahaemolyticus*. H-NS appears to act as a major repressor of the virulence of this pathogen. Date presented here would promote us to gain a deeper understanding of H-NS-mediated silencing of horizontally acquired virulence loci in *V. parahaemolyticus*.

## Introduction

*Vibrio parahaemolyticus* is a curved, rod-shaped, Gram-negative bacterium, which inhibits in saltwater or in other brackish waters and is a leading cause of seafood-associated diarrhea and gastroenteritis. Worldwide outbreaks of *V. parahaemolyticus*-induced infections occur since 1996, which is linked to a clonal pandemic group with higher levels of virulence relative to other types/groups of *V. parahaemolyticus* (Nair et al., 2007).

The reference pandemic strain RIMD2210633 expresses thermostable direct hemolysin (TDH) and two distinct type III secretion systems T3SS1 (VP1656-VP1702) and T3SS2 (VPA1321-1731), and the T3SS2 locus and two copies of *tdh* (*tdh2*:VPA1314 and *tdh1*:VPA1378) are located in the pathogenicity island named Vp-PAI (VPA1312-1398) (Makino et al., 2003). Both T3SS1 and Vp-PAI loci have complex transcriptional organization, each of which consists of at least 10 putative operons (Makino et al., 2003). TDH, T3SS1, and T3SS2 are the major determinants of hemolytic, cytotoxic, and enterotoxic activities, respectively (Hiyoshi et al., 2010). *tdh2* is expressed at a much high level than *tdh1* and thereby is the predominant determinant of TDH activity (Okuda and Nishibuchi, 1998).

RIMD2210633 harbors two type VI secretion systems T6SS1 (VP1386-1414) and T6SS2 (VPA1024-1046) (Makino et al., 2003). T6SS1 has the anti-bacterial activity and enhances the fitness of *V. parahaemolyticus* when competing with other bacterial populations in environments (Salomon et al., 2013, 2014a). Both T6SS1 and T6SS2 contribute to the adhesion to cultured cell monolayers (Yu et al., 2012). All the above gene loci are most likely acquired through horizontal gene transfer at the speciation of *V. parahaemolyticus* or during intraspecies microevolution (Makino et al., 2003; Han et al., 2008; Xiao et al., 2011). The T6SS2 locus is composed of three putative operons VPA1027-1024, VPA1043-1028, and VPA1044-1046 (Makino et al., 2003).

H-NS is a nucleoid-associated DNA-binding protein widely presented in Gram-negative bacteria. Although primarily being as a dimer at low concentrations, H-NS multimerizes into higher order complexes in cells to precede its DNA-binding and thereby to form bridges between adjacent DNA helices to silence target gene transcription (Fang and Rimsky, 2008). H-NS has a preference for A+T-rich DNA sequences so as to repress the transcription of horizontally transferred genes in a process called xenogeneic silencing (Fang and Rimsky, 2008). Bacteria also evolve to derepress H-NS-silenced foreign genes to benefit from their expression, which involves an array of anti-silencing mechanisms at least including prevention of H-NS polymerization along target DNA, displacement of H-NS from target promoter, and modification of target promoter conformation (Stoebel et al., 2008).

It has been previously shown that *V. parahaemolyticus* H-NS serves as a repressor of T6SS1 and that the activation of surface sensing as well as the high salt conditions alleviates the H-NS-dependent repression (Salomon et al., 2014b). Another preliminary report (Kodama et al., 2010b) shows that the H-NS-mediated transcriptional repression of *exsA*, which encodes the transcriptional activators of T3SS1 (Zhou et al., 2008). The present work shows that H-NS act as a major repressor of the virulence of *V. parahaemolyticus* through directly acting on three virulence loci T3SS1, Vp-PAI, and T6SS2.

## Experimental procedure

### Bacterial strains

The wild-type (WT) *V. parahaemolyticus* strain RIMD 2210633 was isolated from a patient with traveler's diarrhea in Japan in 1996 (Makino et al., 2003). To generate the *hns* null mutant (Δ*hns*), the base pairs (bps) 17–396 (referred to as the target deletion region) of the *hns* coding region was deleted from WT using the suicide plasmid pDS132 (Philippe et al., 2004) by introducing the homologous recombination (Casselli et al., 2008; Hiyoshi et al., 2010). Two DNA fragments (540 and 618 bp in length, respectively) flanking the target deletion region were amplified by PCR, purified, and used as the templates to create a 1158 bp deletion allele that was subsequently inserted between the *Pst* I and *Sph* I sites of pDS132. All the primers used in the present work were listed in Table S1. Upon being verified by DNA sequencing, the recombinant vector (containing the 1158 bp deletion allele, the *sacB* gene conferring sensitivity to sucrose, and a chloramphenicol resistance gene) was introduced into *Escherichia coli* S17-1(*pir*), and then transferred into WT by conjugation. The recombinant vector was able to replicate in S17-1 producing the π protein (the product of *pir* gene), but not in *V. parahaemolyticus*. The *V. parahaemolyticus* cells in which the plasmid was integrated into the chromosomal DNA through homologous recombination (the first step of allelic exchange) were screened with resistance to chloramphenicol. The candidate cells were then spread onto the agar plate containing 1% polypepton, 0.5% yeast extract, 30 mM NaCl, 55 mM KCl, 10% sucrose, and 2.5% agar. The mutant strain with the excision of integrated plasmid through the second round of allelic exchange was selected with resistance to 10% sucrose and sensitivity to chloramphenicol, and further verified by PCR.

A PCR-generated DNA fragment composed of the *hns* coding region together with a upstream synthetic ribosome binding site (RBS) was cloned between the *Xba* I and *HinD* III sites of the pBAD33 vector (Guzman et al., 1995) harboring an arabinose P_BAD_ promoter and a chloramphenicol resistance gene. Upon being verified by DNA sequencing, the recombinant plasmid pBAD33-*hns* was introduced into Δ*hns* through electrotransformation, yielding the complemented mutant strain Δ*hns*/pBAD33-*hns*. In addition, the empty vector pBAD33 was introduced into WT or Δ*hns* to generate WT/pBAD33 or Δ*hns*/pBAD33, respectively.

### Bacterial growth conditions

For the general *V. parahaemolyticus* cultivation and maintenance, bacteria were cultured in the HI broth [2.5% Bacto heart infusion (BD Bioscience)] or on the HI plate (2.5% Bacto heart infusion, and 1.5% bacteriological grade agar) at 37°C. For the long term storage, bacteria were stored in the HI broth with the addition of 30% glycerol at −85°C. Chloramphenicol or rabinose was added at 10 μg/ml or 0.1%, respectively, when needed. We used a design of two-round precultivation of bacterial cells: firstly, the glyceric stock of bacteria was inoculated into 15 ml of HI broth for growing at 37°C with shaking at 200 r/min for 12–14 h to enter the stationary growth phase; secondly, the resulting cell cultures were 50-fold diluted into 15 ml of HI broth with the addition of 0.1% arabinose and allowed to grow under the above conditions to reach an OD_600_ value of about 1.4–1.6 (at the mid-exponential growth phase), and then the cell culture was diluted with the HI broth to an OD_600_ value of about 1.4.

### Murine infection model

The precultivated bacterial cells were washed twice with the PBS buffer (pH7.2), and then subjective to serial 10-fold dilutions with PBS. Appropriate dilutions were plated onto the HI plates to calculate the numbers of colony-forming unit (CFU). For each strain tested, 0.1 ml of the 10^9^ CFU/ml bacterial suspension were inoculated intraperitoneally into each of the 15 female BALB/c mice that were 25–28 days old (Hiyoshi et al., 2010), after which the numbers of mice killed at specified times were calculated.

### Cytotoxicity assays

The cytotoxic assays were performed as described previously with some modifications (Hiyoshi et al., 2010). The precultivated bacterial cells were washed and serially 10-fold diluted with the pre-warmed Dulbecco's modified Eagle's medium (DMEM) lacking phenol red for CFU measurement and infection. RAW 264.7 cells were infected with 10^6^ CFU of bacteria for 3 h at a multiplicity of infection (MOI) of 2.5. After infection, the release of lactate dehydrogenase (LDH) into the medium was quantified with a CytoTox96 kit (Promega) according to the manufacturer's instructions.

### Determination of hemolytic activity

The hemolytic activity assay was performed as described previously (Ono et al., 2006) with slight modifications. Rabbit erythrocytes (RECs) were diluted into DMEM lacking phenol to a 5% (v/v) final concentration (designated as 5% REC-DMEM). The precultivated bacterial cells were washed and serially 10-fold diluted with the HI broth for CFU measurement and infection. 10 μl of the 10^8^ CFU/ml bacterial suspension was mixed with 500 μl of 5% REC-DMEM, and centrifuged at 2500 × g for 1 min. The HI broth was used as the negative control to infect RECs for background subtraction. After 3.5 h of incubation at 37°C without shaking, the pellet was gently resuspended to facilitate the release of hemoglobin. Cells were repelleted at 12,000 × g for 1 min, and the supernatant was monitored for the presence of released hemoglobin by determining the OD_570_ values.

### Rabbit ileal loop test

The procedures for the rabbit ileal loop test was modified from those described previously (Nishibuchi et al., 1992). Briefly, four loops (10 cm each in length) were placed in the small intestine of each of six rabbits. The precultivated bacterial cells were washed and diluted with the HI broth for CFU measurement and infection. 1 ml of the 10^9^ CFU/ml bacterial suspension was injected into each ileal loop. The HI broth was injected as the negative control for background subtraction. The rabbits were sacrificed by the venous air embolism at 14 h after injection, and the fluid accumulation (in milliliters) was calculated as the amount of accumulated fluid of each ligated rabbit ileal loop. Isoflurane was employed for the inhalation anesthesia of rabbits before each surgery. All the animal experiments were approved by the Committee on Animal Research of the Academy of Military Medical Sciences and carried out in accordance with the approved guideline.

### RNA isolation and quantitative reverse transcription PCR (qRT-PCR)

The precultivated bacterial cells were 1000-fold diluted into 15 ml of HI broth, and allowed to grow at 37°C with shaking at 200 r/min to an OD_600_ value of about 0.4 for bacterial harvest. Total RNAs were extracted using the TRIzol Reagent (Invitrogen). RNA quality was monitored by agarose gel electrophoresis, and RNA quantity was determined by spectrophotometry. The contaminated DNA in the RNA samples was removed by using the Amibion's DNA-free™ Kit. cDNAs were generated by using 5 μg of RNA and 3 μg of random hexamer primers. Real-time PCR was performed through the LightCycler system (Roche) together with the SYBR Green master mix (Zhan et al., 2008). Based on the standard curve of 16S rRNA expression for each RNA preparation, the relative mRNA levels were determined with the ΔCt values by the classic ΔCt method. 16S rRNA gene was used to normalize.

### Primer extension assay

As described previously (Zhang et al., 2013a,b), a 5′−^32^P-labeled oligonucleotide primer complementary to a portion of the RNA transcript of each indicated gene was employed to synthesize cDNAs from total RNA template using a Primer Extension System (Promega). If different *V. parahaemolyticus* strains were involved in a single experiment, equal amounts of total RNA were used as the starting materials. Sequence ladders were prepared with the same 5′−^32^P-labeled primers using AccuPower and Top DNA Sequencing Kit (Bioneer). Radioactive species were detected by autoradiography. The 5′-terminus of each RNA transcript (i.e., the transcription start) was mapped according to the size of primer extension product, while the relative mRNA levels were determined with the intensities of primer extension product.

### LacZ fusion and β-galactosidase assay

The promoter-proximal DNA region of each indicated gene was amplified by PCR with ExTaq™ DNA polymerase (Takara) using RIMD 2210633 genome DNA as the template. PCR fragments were then cloned between *Sal* I and *EcoR* I or *Xba* I sites of low-copy-number transcriptional *lacZ* fusion vector pHRP309 that harbors a gentamicin resistance gene and a promoterless *lacZ* reporter gene (Zhang et al., 2011). Correct cloning was verified by DNA sequencing. An empty pHRP309 plasmid was also introduced into each strain tested as the negative control. *V. parahaemolyticus* strains transformed with recombinant or empty pHRP309 plasmids were grown as that for RNA isolation to measure the β-galactosidase activity in cellular extracts using a β-Galactosidase Enzyme Assay System (Promega).

### Preparation of 6× His-tagged H-NS (His-H-NS) protein

The entire coding region of hns of strain RIMD 2210633 was cloned between *BamH*I and *Hind*III sites of plasmid pET28a (Novagen). The recombinant plasmid encoding His-H-NS was transformed into *E. coli* BL21λ DE3 cells, and grown in the Luria-Bertani (LB) broth at 37°C with shaking at 200 r/min for 4–5 h. The resulting culture was diluted 1/100 into 200 to 300 ml of fresh LB broth, and grown under the above conditions to an OD_600_ of about 0.5. The culture was shifted to 18°C for 1 h, and then induced with 1 mM IPTG for 16 to 18 h with shaking at 100 rpm. Cells were collected by centrifugation and frozen at −60°C. The pellet was resuspended in 10 ml of 50 mM sodium phosphate buffer, pH 7.4, 500 mM NaCl, and 5 mM imidazole. Cells were disrupted using a cell cracker, and the insoluble material was pelleted by centrifugation at 12,000 rpm. The clarified supernatant was applied to a 3 ml Ni-NTA Agarose Column (Qiagen), and the overproduced protein was purified under native conditions. Fractions from a homogenous peak were pooled, and the final preparation was dialyzed against 10 mM Tris HCl, pH 7.4, 10 mM NaCl, 1 mM EDTA, 0.1 mM DTT, and 20% glycerol. The purified protein was stored at −60°C, and the protein purity was verified by SDS-PAGE.

### Electrophoresis mobility shift assay (EMSA)

The promoter-proximal DNA region of each indicated gene was amplified by PCR. For EMSA (Ishikawa et al., 2009; Lesic et al., 2009), the 5′ ends of DNA were labeled using [γ−^32^P] ATP and T4 polynucleotide kinase. DNA binding was performed in a 10 μl reaction volume containing binding buffer [1 mM MgCl_2_, 0.5 mM EDTA, 0.5 mM DTT, 50 mM NaCl, 10 mM Tris-HCl (pH 7.5) and 0.05 mg/ml poly-(dI-dC)], labeled DNA (1000 to 2000 c.p.m/μl), and increasing amounts of His-H-NS. Three controls were included in each EMSA experiment: (1) cold probe as specific DNA competitor (the same promoter-proximal DNA region unlabeled), (2) negative probe as non-specific DNA competitor (the unlabeled coding region of the 16S rRNA gene), and (3) non-specific protein competitor [rabbit anti-F1-protein polyclonal antibodies]. The F1 protein is the protective antigen from *Yersinia pestis* (Andrews et al., 1996). After incubation at room temperature for 30 min, the products were loaded onto a native 4% (w/v) polyacrylamide gel, and electrophoresed in 0.5× TBE buffer for about 50 min at 220 V. Radioactive species were detected by autoradiography after exposure to Kodak film at −70°C.

### DNase I footprinting

For DNase I footprinting (Ishikawa et al., 2009; Lesic et al., 2009), the promoter-proximal DNA regions with a single ^32^P-labeled end were PCR amplified with either sense or antisense primer being end-labeled. The PCR products were purified using the QiaQuick columns (Qiagen). Increasing amounts of His-H-NS were incubated with the purified, labeled DNA fragment (2–5 pmol) for 30 min at room temperature, in a final 10 μl reaction volume containing the binding buffer used in EMSA. Before DNA digestion, 10 μl of Ca^2+^/Mg^2+^ solution (5 mM CaCl_2_ and 10 mM MgCl_2_) was added, followed by incubation for 1 min at room temperature. The optimized RQ1 RNase-Free DNase I (Promega) was then added to the reaction mixture, and the mixture was incubated at room temperature for 40–90 s. The reaction was quenched by adding 9 μl of stop solution (200 mM NaCl, 30 mM EDTA, and 1% SDS), followed by incubation for 1 min at room temperature. The partially digested DNA samples were extracted with phenol/chloroform, precipitated with ethanol, and analyzed in 6% polyacrylamide/8 M urea gel. Protected regions were identified by comparison with sequencing ladders. The templates for sequencing were the same as DNA fragments for DNase I footprinting. Radioactive species were detected as above.

### Experimental replicates and statistical methods

For the phenotype, LacZ fusion, and RT-PCR assays, experiments were performed with at least three independent bacterial cultures, and the values were expressed as mean ± standard deviation. Paired Student's *t*-test was performed to determine statistically significant differences; *P* < 0.01 was considered to indicate statistical significance. For the primer extension, EMSA, and DNase I footprinting assays, representative data from at least two independent biological replicates were shown.

## Results

### Effect of H-NS on mutlple virulence phenotypes

**Figure 1:**
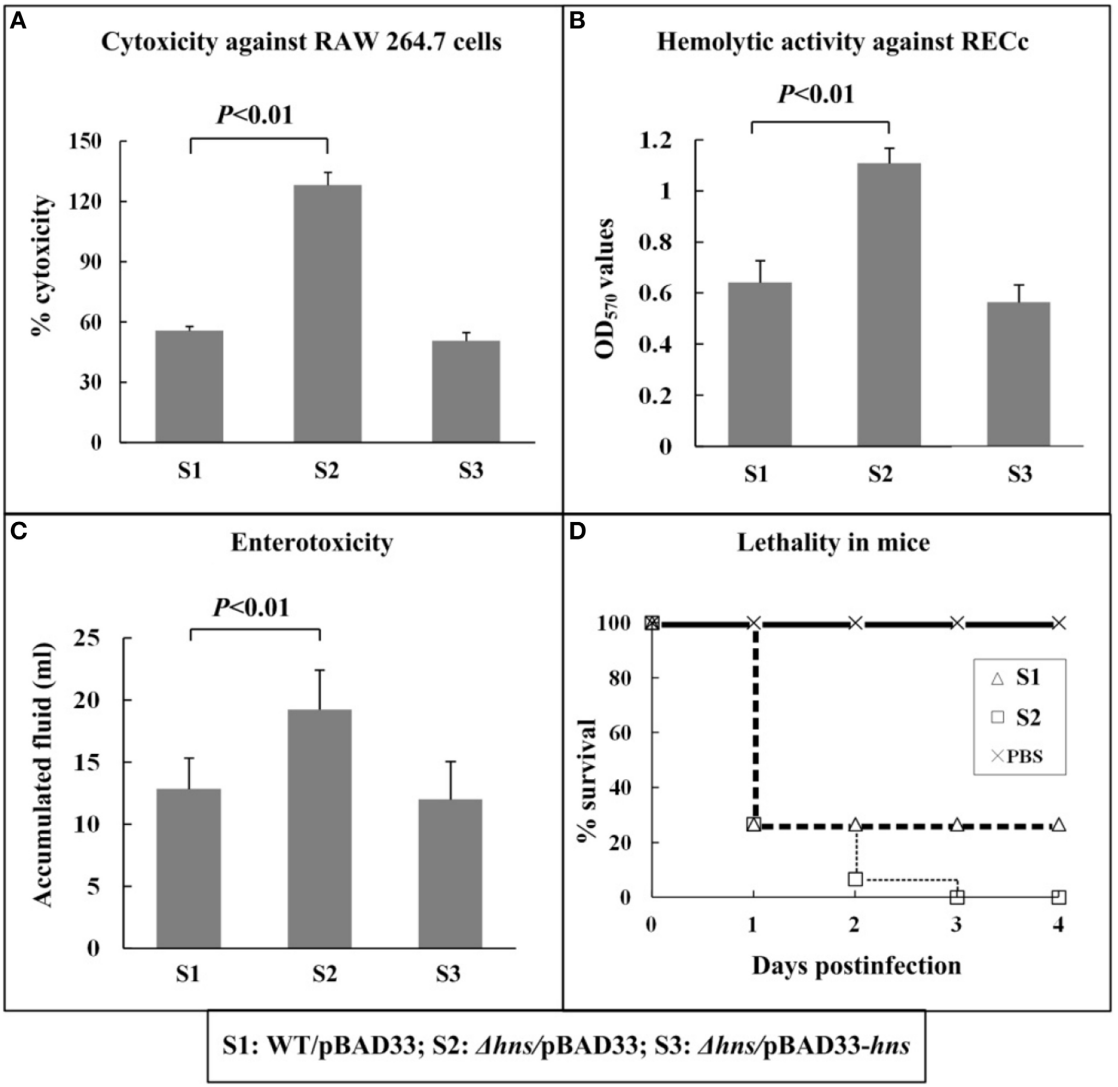
**Involvement of H-NS in virulence**. The cytotoxicity against cultured RAW 264.7 cells **(A)** was evaluated in terms of the release of LDH. The hemolytic activity against RECs **(B)** was evaluated by detecting the release of hemoglobin. The enterotoxicity was evaluated by determining the fluid accumulation in the ileal loop **(C)**. The murine survival rate after the intraperitoneal infection was measured to determine the lethality in mice **(D)**.

WT/pBAD33, Δ*hns*/pBAD33, and Δ*hns*/pBAD33-*hns* were employed in all the phenotype assays, which would normalize the effects of chloramphenicol and rabinose, when added in cell cultures, on bacterial growth and physiology. First, *V. parahaemolyticus*-induced cytotoxic activity against the macrophage-like cell line RAW 264.7 was evaluated in terms of the release of cytosolic LDH from cultured cells (Figure 1A). When RAW 264 cells were infected with Δ*hns*/pBAD33, the cytotoxicitiy was significantly enhanced compared with that of WT/pBAD33 or Δ*hns*/pBAD33-*hns*. Second, the hemolytic activities against RECs were compared between the above three strains (Figure 1B). The hemolytic activity of Δ*hns*/pBAD33 was significantly higher than that of WT/pBAD33 or Δ*hns*/pBAD33-*hns*. Third, the fluid accumulating activities of the above three strains in rabbit ileal loops were compared to investigate the enterotoxicities of these strains (Figure 1C). Δ*hns*/pBAD33 showed a significant increase in fluid accumulation compared with WT/pBAD33 and Δ*hns*/pBAD33-*hns*. Finally, the survival rates of mice infected intraperitoneally with each of the above three strains were determined (Figure 1D), and the lethality in mice for Δ*hns*/pBAD33 was significantly elevated relative to WT. Δ*hns*/pBAD33-*hns* exhibited a curve of murine survival rates similar to that of Δ*hns*/pBAD33 (data not shown), which might be due to lack of arabinose for efficiently inducing expression of H-NS or that of antibiotics for maintaining replication of pBAD33-*hns* (for *in trans* complementation) during infection in mice. Taken together, H-NS inhibits the cytotoxicitiy, enterotoxicity, hemolytic activity, and mouse lethality of *V. parahaemolyticus*.

### Negative and direct regulation of H-NS on mutlple virulence loci

The putative operons VP1700-1688 and VP1699-1688 (T3SS1 regulation and apparatus) from T3SS1, the putative operons *vtrA* (VPA1332; Vp-PAI regulation), VPA1362-1358 (T3SS1 apparatus) and *tdh2* (TDH) from Vp-PAI, and all the three putative operons from T6SS2 were arbitrarily selected (Table 1), and the first genes of them was subjected to the investigation of H-NS-mediated gene regulation through qRT-PCR, primer extension, LacZ fusion, EMSA, and DNase I footprinting.

**Table 1:** **Direct H-NS targets characterized in this study**.

**Category**	**First gene**		**Operon**		**Cellular function**
	**ID**	**Name**	**ID range**	**Gene range**	
T3SS1	VP1700	*exsB*	VP1700-1688	*exsBAD-vscBCD FGHIJKL*	T3SS1 regulation and apparatus
	VP1699	*exsA*	VP1699-1688	*exsAD-vscBCD FGHIJKL*	
Vp-PAI (T3SS2+TDH)	VPA1332	*vtrA*	VPA1332	*vtrA*	Vp-PAI regulation
	VPA1362	*vopB2*	VPA1362-1358	Not applicable	T3SS2 apparatus
	VPA1314	*tdhA/tdh2*	VPA1314	*tdhA/tdh2*	TDH
T6SS2	VPA1027	*tssD2/hcp2*	VPA1027-1022	Not applicable	T6SS2 apparatus
	VPA1043	*tagH2/fha2*	VPA1043-1028	*tagH_2_-tssJ_2_K_2_L_2_M_2_- tagF_2_G_2_-tssA_2_B_2_C_2-1_C_2-2_ -tagJ_2_E_2_F_2_G_2_H_2_*	
	VPA1044	*tagE2/ppkA2*	VPA1044-1466	Not applicable	

WT and Δ*hns* (but not Δ*hns*/pBAD33-*hns*) were used for gene regulation experiments, due to the facts that Δ*hns*/pBAD33-*hns* and WT/pBAD33 gave very similar phenotypes (see above), and that no change in expression of VP1132 and VP1134 (downstream and upstream of *hns*, respectively) was detected in Δ*hns*/pBAD33-*hns* relative to WT/pBAD33 by using qRT-PCR (data not shown).

**Figure 2:**
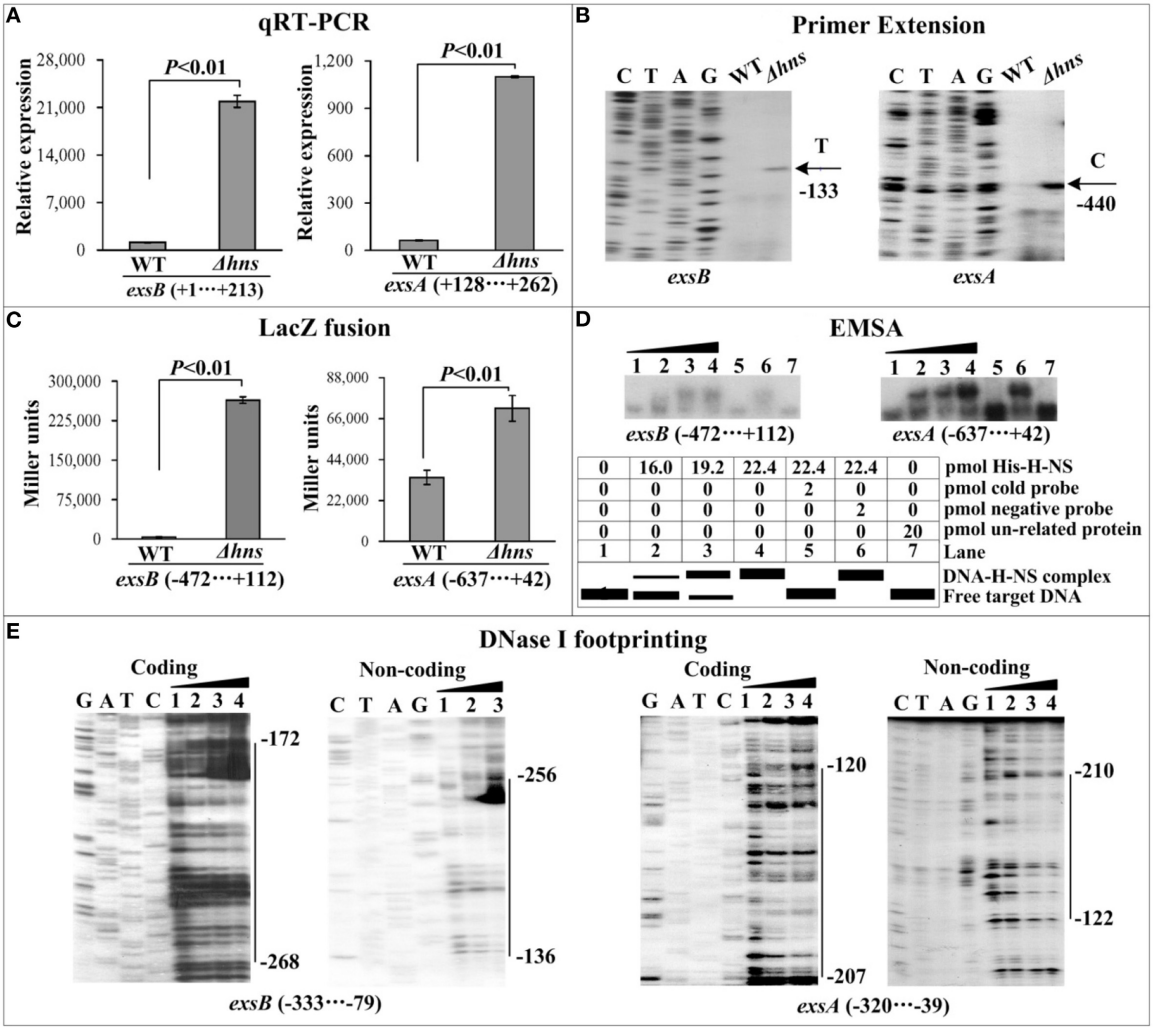
**Regulation of T6SS1 genes by H-NS**. Lanes C, T, A, and G represent the Sanger sequencing reactions. The minus and positive numbers in the brackets or on the right side of the vertical bars indicated the nucleotide positions upstream and downstream of indicated genes. **(A)** qRT-PCR. The relative mRNA levels of each target gene were compared between Δ*hns* and WT. **(B)** Primer extension. An oligonucleotide primer was designed to be complementary to the RNA transcript of each target gene. The primer extension products were analyzed with an 8 M urea-6% acrylamide sequencing gel. The transcriptional starting sites were indicated by arrows with nucleotides and positions. **(C)** LacZ fusion. The target promoter-proximal DNA region was cloned into the *lacZ* transcriptional fusion vector pHRP309 and then transformed into WT or Δ*hns* to determine the promoter activity, i.e., the β-galactosidase activity (miller units) in the cellular extracts. **(D)** EMSA. The radioactively labeled promoter-proximal DNA fragments were incubated with increasing amounts of purified His-H-NS protein and then subjected to 4% (w/v) polyacrylamide gel electrophoresis. If there was the association between His-H-NS and target DNA, the band of free DNA disappeared with increasing amounts of His-H-NS, resulting in a retarded DNA band (representing the DNA-His-H-NS complex) with decreased mobility. The schematic representation of the EMSA design was shown also. **(E)** DNase I footprinting. Labeled coding or non-coding DNA probes were incubated with increasing amounts of purified His-H-NS (Lanes 1, 2, 3, and 4 containing 0, 44.2, 66.3, and 77.3 pmol, respectively) and then subjected to DNase I footprinting assay. The footprint regions were indicated by vertical bars with positions.

**Figure 3:**
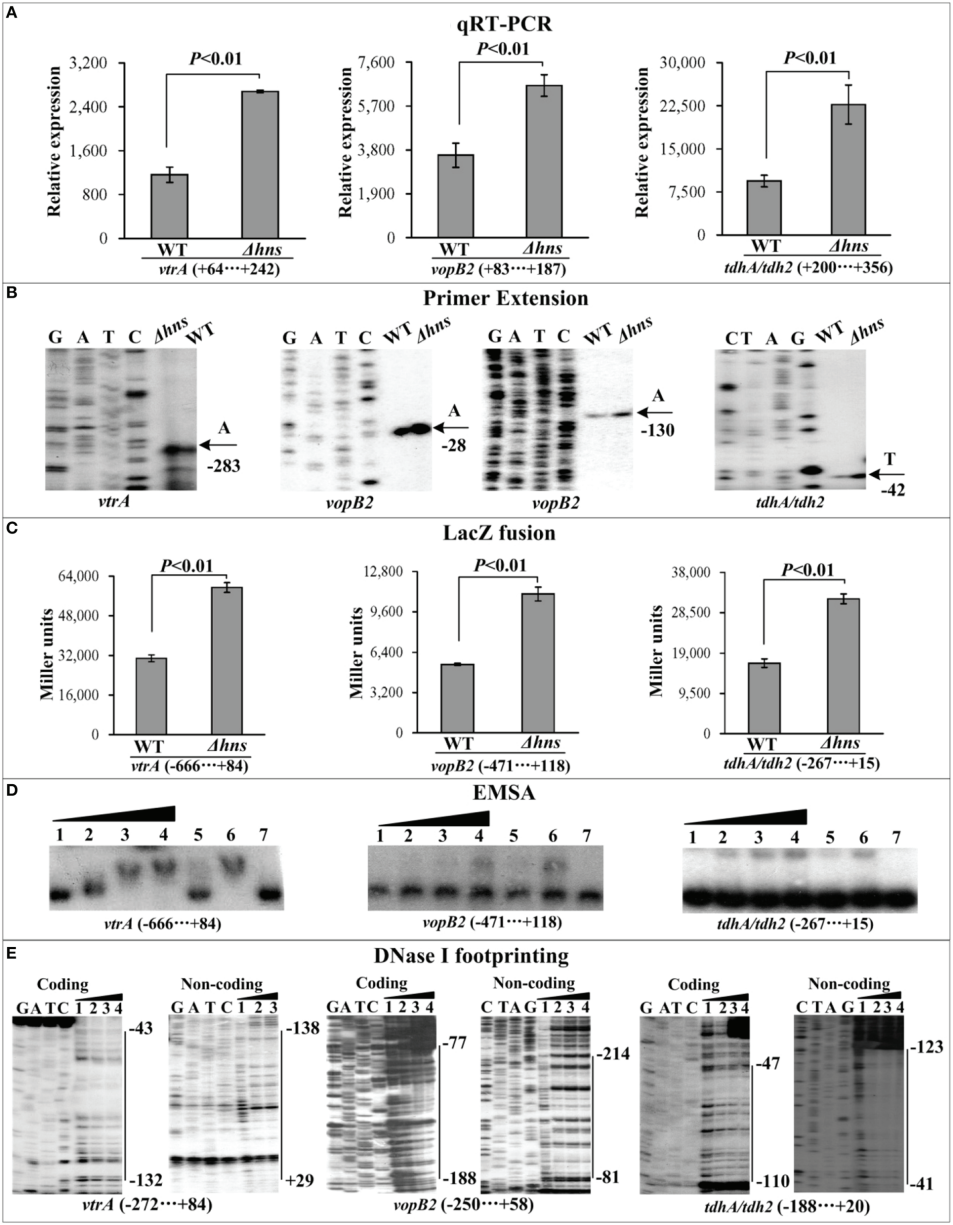
**Regulation of Vp-PAI genes by H-NS**. qRT-PCR **(A)**, primer extension **(B)**, LacZ fusion **(C)**, EMSA **(D)**, and DNase I footprinting **(E)** were performed as Figure 2.

**Figure 4:**
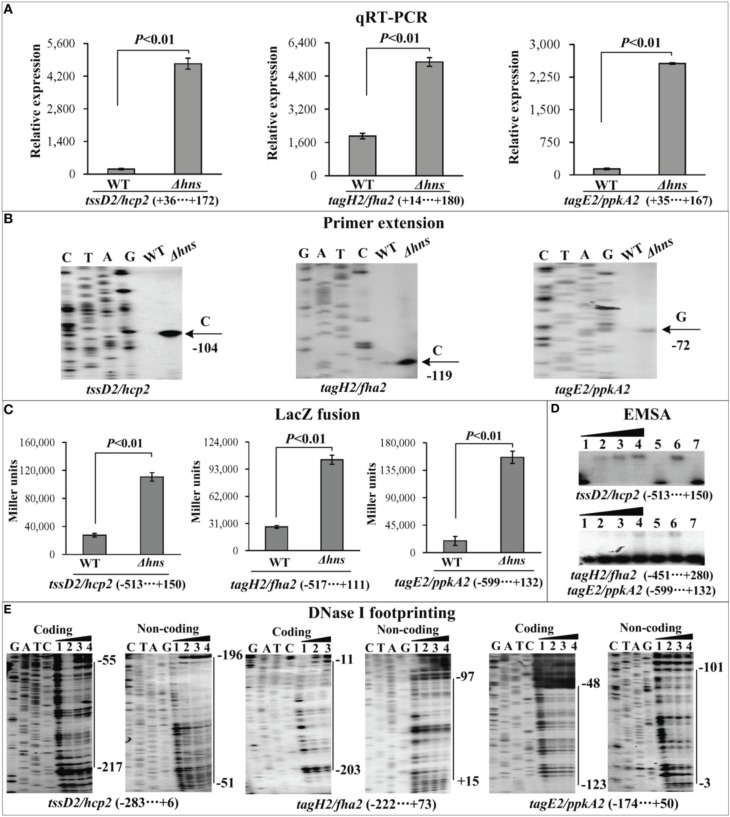
**Regulation of T6SS2 genes by H-NS**. qRT-PCR **(A)**, primer extension **(B)**, LacZ fusion **(C)**, EMSA **(D)**, and DNase I footprinting **(E)** were performed as Figure 2.

The mRNA levels of each target gene in Δ*hns* and WT were determined by qRT-PCR (Figures 2A, 3A, 4A) and primer extension (Figures 2B, 3B, 4B). The results from both kinds of assay showed that the mRNA level of each target was significantly enhanced in Δ*hns* relative to WT. To test action of H-NS on the promoter activity of each target gene, the fusion promoter consisting of a promoter-proximal region of each target gene together with the promoterless *lacZ* was constructed in pHRP309, and then transformed into WT and Δ*hns*, respectively (Figures 2C, 3C, 4C); the relative promoter activity (the β-Galactosidase activity) of each target gene in Δ*hns* was significantly higher than that in Δ*hns*. The above results disclosed the negative regulation of each target gene by H-NS.

For EMSA (Figures 2D, 3D, 4D), a 250–800 bp promoter-proximal DNA fragment for each target gene was radioactively labeled, incubated with a purified His-H-NS protein, and then subjected to native gel electrophoresis. His-H-NS was able to bind to each target DNA fragment in a dose-dependent manner. By contrast, His-H-NS at all tested amounts could not bind to a partial coding region of the 16S rRNA gene as the negative control (data not shown). Subsequent DNase I footprinting (Figures 2E, 3E, 4E) showed that His-H-NS protected a single region within each target promoter-proximal DNA fragment against DNase I digestion in a dose-dependent manner. Taken together, H-NS negative controls the transcription of the above target operons through binding to their promoter-proximal regions.

## Discussion

The major human-pathogenic species in the *Vibrio* genus include *V. cholerae* (the causative agent of cholera), *V. vulnificus* (causing cellulitis and septicemia), and *V. parahaemolyticus*. *V. cholerae* expresses two major virulence factors, toxin coregulated pilus (TCP, encoded by the *tcp* operon) and cholera toxin (CT, by the *ctx* operon), both of which are required for the infection of intestinal mucosa (Wang et al., 2012). Later in infection, *V. cholerae* inhibits the expression of TCP and CT, but allows that of motility and hemagglutinin (HA)/protease (encoded by *hapA)*, which facilitates the detachment from intestinal mucosa (Wang et al., 2012). H-NS binds to the promoter-proximal regions of *tcp*, *ctx*, *hapA*, and *flrA* (encoding a transcriptional activator of motility) to repress their transcription (Stonehouse et al., 2008, 2011). H-NS sites overlap with the sites of two transcriptional regulators ToxT and IHF at the promoter regions of *tcp* and *ctx*, and transcriptional silencing of *tcp* or *ctx* by H-NS is antagonized by ToxT and IHF (Stonehouse et al., 2008, 2011). RpoS acts to attenuate H-NS-mediated silencing *hapA* and *flrA* indirectly by enhancing the expression of IHF and also directly by promoting transcription initiation resistant to H-NS (Wang et al., 2012).

The *rtxA1* gene encodes a repeat-in-toxin protein essential for the virulence of *V. vulnificus*, and similarly its transcription is repressed by H-NS, which is subjected to the derepression of the HlyU regulator by direct contact of the upstream DNA region of *rtxA1* (Liu et al., 2009).

The present work demonstrates that *V. parahaemolyticus* H-NS occupies the promoter-proximal regions of two or more operons in each of the three virulence loci T3SS1, Vp-PAI, and T6SS2 to repress their transcription, and that H-NS inhibits multiple virulence phenotypes including at least cytotoxicitiy, enterotoxicity, hemolytic activity, and mouse lethality. Because of H-NS action on *exsA* and *vtrA*, which encode the transcriptional activators of T3SS1 and Vp-PAI (Zhou et al., 2008; Kodama et al., 2010a), respectively, it can be deduced that all the T3SS1 and Vp-PAI genes are under negatively controlled by H-NS. In addition, H-NS recognizes all the three operons harbored in the T6SS2 locus. H-NS appears to act as a major repressor of the virulence of *V. parahaemolyticus*.

**Figure 5:**
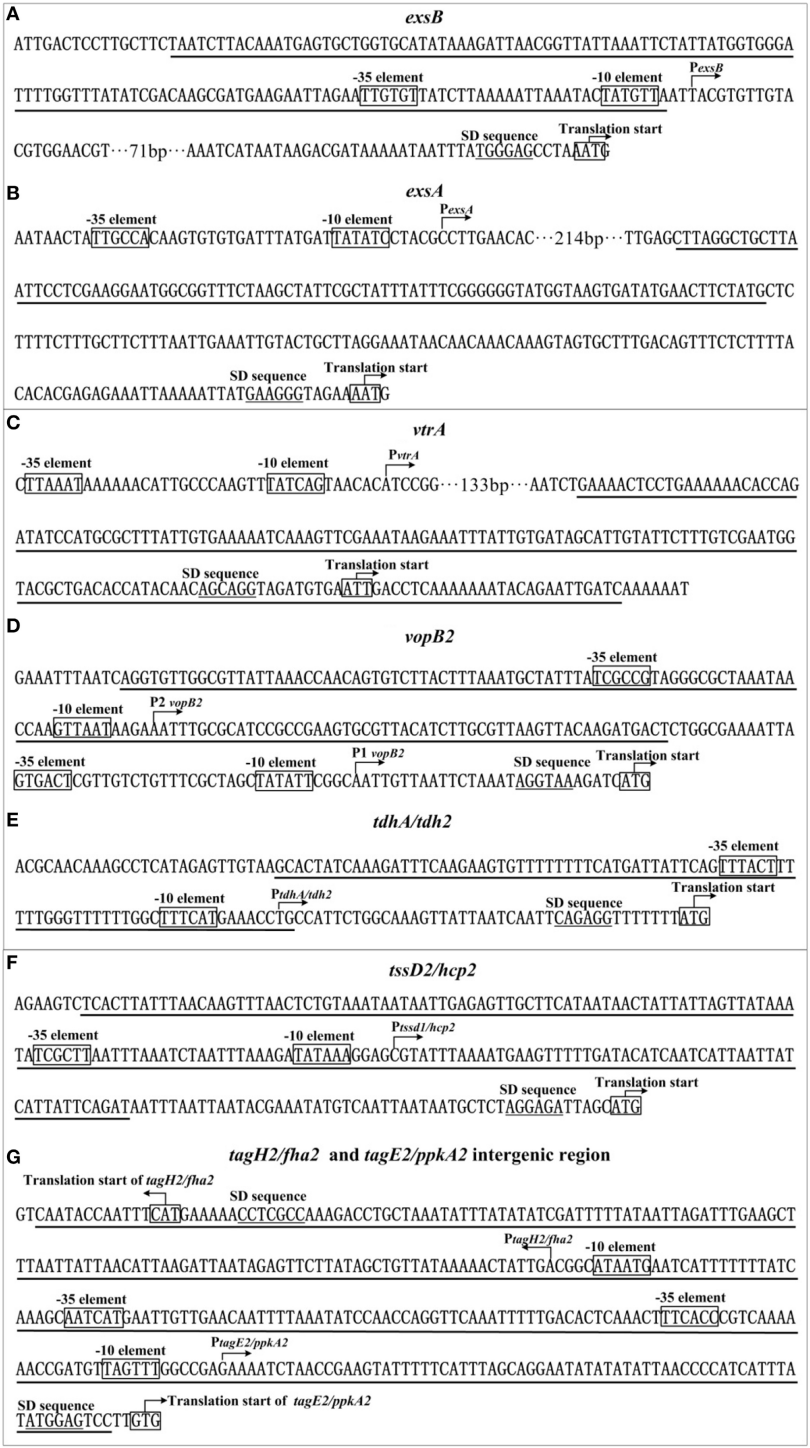
**Organization of promoter-proximal DNA regions**. The promoter-proximal DNA regions of indicated genes (**A**: exsB; **B**: exsA; **C**: vrtA; **D**: vopB2; **E**: tdh2; **F**: hcp2; **G**: tagH2, and tagE2 intergenic) were derived from RIMD 221063. Underlined nucleotide sequences are the H-NS sites.

Primer extension detects a single H-NS-dependent promoter (a single transcriptional start, and corresponding −10 and −35 elements) for each target operon. The single footprint region determined by DNase I footprinting is considered as the H-NS binding site for each target promoter-proximal region. The H-NS sites range from 91 to 332 bp in length, and have the G+C contents of from 20 to 42%, which are lower than the mean value of 45.4% of the genome. The structural organization of each H-NS-dependent promoter can be reconstructed based on the collection of translation/transcription starts, core promoter −10 and −35 elements for RNA polymerase (RNAP) association, Shine-Dalgarno (SD) sequences for ribosomal binding, and H-NS sites (Figure 5). Notably, each of the detected H-NS sites overlaps the core promoter region (−10 and −35 elements) of the corresponding target operon, and thus H-NS is thought to silence gene transcription by directly interfering with RNAP action. Nevertheless, potential anti-silencing factors of the above H-NS-repressed virulence loci, which might act like ToxT, IHF, and HlyU in other *Vibrio* species, need to be characterized.

### Conflict of interest statement

The Associate Editor, Beiyan Nan, declares that despite having collaborated with author Dongsheng Zhou on the same Research Topic, the review process was handled objectively and no conflict of interest exists. The authors declare that the research was conducted in the absence of any commercial or financial relationships that could be construed as a potential conflict of interest.
