# Supplementary material for: H-NS is a repressor of major virulence gene loci in Vibrio parahaemolyticus
Source: Front Microbiol. 2014 Dec 12;5:675. doi: 10.3389/fmicb.2014.00675 (PMC4264476; doi:10.3389/fmicb.2014.00675)
Supplement: Supplementary file 1 [file Table1.DOC]

**Table S1 Primers used in this study**

| **Target** | | **Primers (forward/reverse, 5′-3′)** |
| --- | --- | --- |
| **Construction of mutant** | | |
| *hns* | | GTGACTGCAGACTTATGATGAGAACCAATGC/ CAAGAACGATTAGATTAGGAATTAGTCAGCTCTGACATAACG |
|  |  | CGTTATGTCAGAGCTGACTAATTCCTAATCTAATCGTTCTTG/ GTGAGCATGCAGAGTGGGCTGATATGGTG |
|  |  | GTGACTGCAGACTTATGATGAGAACCAATGC/GTGAGCATGCAGAGTGGGCTGATATGGTG |
| **Construction of complemented mutant** | | |
| *hns* | GATCCCGGGAGGAGGAATTCACCATGTCAGAGCTGACTAAAACAC/ GACGTCGACTTAGATTAGGAAATCGTCTAG | |
| **Protein expression** | | |
| *hns* | | AGCGGGATCCATGTCAGAGCTGACTAAAACAC/ AGCGAAGCTTTTAGATTAGGAAATCGTCTAG |
| **qRT-PCR** | | |
| *exsB* | | ATGAAAAGCAGTAAGTGGGC/CTGAGAAGCAACAGTAAGAC |
| *exsA* | | ACAATGGTCAATTACGAGTTC/CAATGATAGATGAGTGATAATTCC |
| *vtrA* | | AGTCTAGGCTCACAAGATCG/AAATGGGCTCTGATGTTACG |
| *vopB2* | | ACCAGCCTCAGCAACAAGC/CTTTCACGAATACTACGC |
| *tdhA/tdh2* | | ATGTAAAAAGAAAACCGTACA/AACACAGCAGAATGACCGTG |
| *tssd2/hcp2* | | TAAAGGTGAAGCGACAGCG/AATCATATAGGCGTGTTGC |
| *tagH2/fha2* | | TGACCATAACGAGTTTCCAC/TTTAATCAATTCGCCGTGAG |
| *tagE2/ppkA* | | ATAGCAGCGATAGCGGAG/TTTGAGACAGTTTTGTATCC |
| **Primer extension** | | |
| *exsB* | | /GTCTTATTATGATTTATTTTTACAC |
| *exsA* | | /TGACTTTGTTTTTATATTAG |
| *vtrA* | | /GAGATTCGTAGCGTATAAGTGC |
| *vopB2* | | /TAGCTTGCCCCCACAGAG |
| *tdhA/tdh2* | | /GCAAAATATCGGTACTTCA |
| *tssd2/hcp2* | | /CTGCATGCTAATCTCCTAGAGC |
| *tagH2/fha2* | | /GATTTGAAGCTTTAATTATTAACAT |
| *tagE2/ppkA2* | | /CCGCTATCGCTGCTATTT |
| **LacZ fusion** | | |
| *exsB* | | ATATGTCGACATTGTCCGTCAAATGCAGTTC/TTTTGAATTC CATATACATTCGCTTGGCTCTG |
| *exsA* | | GCGCGTCGACTAGAAACAAAAAAGTAATCG/GCGCGAATTCAGAGCCGACCGTTTCTGTG |
| *vtrA* | | GCGCGTCGACTACGCTTCCAATAATCACC/GCGCGAATTCCCGATCTTGTGAGCCTAGAC |
| *vopB2* | | GCGGTCGACGCGTACTAAGTGATGAAGAG/GCGTCTAGACAACAGAACCACTTTCAGC |
| *tdhA/tdh2* | | GCGCGTCGACAATTCACGACGAATCGGAG/GCGCGAATTCATATCGGTACTTCATAAA |
| *tssd2/hcp2* | | GCGCGTCGACTATTACCTTACTTGCCTCTCGG/GCGCGAATTCTGCTTCACGGTCCATTGC |
| *tagH2/fha2* | | GCGCGTCGACTTTGTTGATAGGTGGTATTGTG/ATATGAATTCTGAGCGTCCGAAGGTTAC |
| *tagE2/ppkA2* | | GCGCGTCGACGGGACAAAGCAAGCTCATTC/ATATGAATTCAGCGGAGTCTTGTTTATTAACG |
| **EMSA** | | |
| *exsB* | | ATTGTCCGTCAAATGCAGTTC/CATATACATTCGCTTGGCTCTG |
| *exsA* | | TAGAAACAAAAAAGTAATCG/AGAGCCGACCGTTTCTGTG |
| *vtrA* | | TACGCTTCCAATAATCACC/CCGATCTTGTGAGCCTAGAC |
| *vopB2* | | GCGTACTAAGTGATGAAGAG/CAACAGAACCACTTTCAGC |
| *tdhA/tdh2* | | AATTCACGACGAATCGGAG/ATATCGGTACTTCATAAA |
| *tssd2/hcp2* | | TATTACCTTACTTGCCTCTCGG/TGCTTCACGGTCCATTGC |
| *tagH2/fha2* | | TTTGTTGATAGGTGGTATTGTG/TGAGCGTCCGAAGGTTAC |
| *tagE2/ppkA2* | | GGGACAAAGCAAGCTCATTC/AGCGGAGTCTTGTTTATTAACG |
| **DNase I footprinting** | | |
| *exsB* | | GTTTATCAATTTTGGTTGTTAG/CGGCTTATATTTATTCTAC |
| *exsA* | | AGTCAGAGTTTAAATAGCA/AAAGAGAAACTGTCAAAGCAC |
| *vtrA* | | CCTCGATACTAATAAATCACATG/CCGATCTTGTGAGCCTAGAC |
| *vopB2* | | GGAAGTTGTTGAAAGAGTC/TAGCTTGCCCCCACAGAG |
| *tdhA/tdh2* | | CCGCTTGAGGAATCACAG/GCAAAATATCGGTACTTCA |
| *tssd2/hcp2* | | GCAATTCTTAATGGTAAGACG/CTGCATGCTAATCTCCTAGAGC |
| *tagH2/fha2* | | CTTCGGTTAGATTTTCTCG/ATTCAAACACAAACTCACTCTC |
| *tagE2/ppkA2* | | AAAGCAATCATGAATTGTTGAAC/CCGCTATCGCTGCTATTT |
